# Responsiveness of the Various Short-Form Versions of the Knee Injury and Osteoarthritis Outcome Score Between 2 and 5 Years After Anterior Cruciate Ligament Reconstruction

**DOI:** 10.1177/23259671241236513

**Published:** 2024-03-22

**Authors:** Kate E. Webster, Haydn J. Klemm, Timothy S. Whitehead, Cameron J. Norsworthy, Julian A. Feller

**Affiliations:** † School of Allied Health, Human Services and Sport, La Trobe University, Melbourne, Australia; ‡ OrthoSport Victoria, Epworth HealthCare, Melbourne, Australia; § Faculty of Medicine, Monash University, Melbourne, Australia; ‖ Eastern Health, Box Hill, Australia; Investigation performed at OrthoSport Victoria and La Trobe University, Melbourne, Australia

**Keywords:** ACL, knee, KOOS, patient-reported outcomes

## Abstract

**Background:**

Various short-form versions of the Knee injury and Osteoarthritis Outcome Score (KOOS) have been developed in an attempt to minimize responder burden. However, the responsiveness of these short-form measures in patients who have undergone anterior cruciate ligament (ACL) reconstruction has not been compared at midterm follow-up.

**Purpose:**

To determine the responsiveness of 3 short-form versions of the KOOS (KOOS-12, KOOS-Global, and KOOS-ACL) in patients who have undergone ACL reconstruction.

**Study Design:**

Cohort study (diagnosis); Level of evidence, 3.

**Methods:**

In 276 patients (149 male, 127 female), we administered the KOOS and a measure of overall knee function at both 2 and 5 years after ACL reconstruction. From the full KOOS, the following short-form versions were calculated: KOOS-12, KOOS-Global, and KOOS-ACL. Responsiveness was assessed using several distribution and anchor-based methods for each of the short-form versions. From distribution statistics the standardized response mean (SRM) and smallest detectable change (SDC) were calculated. Using the anchor-based method, the minimally important change (MIC) that was associated with an improvement in knee function was determined using receiver operating characteristic (ROC) analysis.

**Results:**

High ceiling effects were present for all measures. KOOS-Global scores increased significantly over time, whereas KOOS-12 and KOOS-ACL did not change. The KOOS-Quality of Life (QOL) subscale, which can be derived from both KOOS-Global and KOOS-12, also increased significantly between assessments. Both these increases were associated with a small (0.2-0.3) SRM. The MIC was smallest for KOOS-Global (3.2 points) and largest for KOOS-QOL (9.4 points), and, for all measures, the MIC was larger than the SDC at a group level. KOOS-Global was the only measure for which the mean difference between the 2- and 5-year assessments exceeded both the SDC (group level) and the MIC.

**Conclusion:**

Of the 3 short-form versions of the KOOS currently available, the KOOS-Global had the greatest responsiveness to change between the 2- and 5-year assessments after ACL reconstruction. High ceiling effects were present for all versions.

The Knee injury and Osteoarthritis Outcome Score (KOOS) is a measure used frequently after anterior cruciate ligament (ACL) injury and reconstruction to assess a patient's opinion about their knee and associated problems.^24^ However, with 42 items, it has been critiqued for its length and inferior psychometric properties compared with other available measures such as the International Knee Documentation Committee Subjective Knee Form.^12^ Nonetheless, KOOS is the primary patient-reported outcome measure for several ACL registries.^9,14,17^

To overcome some of the limitations associated with use of the full KOOS, several short-form versions have been developed that may be applicable for ACL-injured patients. KOOS-Global is an 11-item version developed by aggregating scores from the KOOS-Joint Replacement short form and the 4 KOOS-Quality of Life (KOOS-QOL) subscale questions.^13,16^ Item selection and validation conducted in patients undergoing ACL reconstruction found that KOOS-Global had good responsiveness between preoperative and 2-year postoperative assessments.^13^ It has been suggested that this measure may be able to capture the transition between an ACL-injured knee and osteoarthritic knee, therefore providing a single outcome measure for use over time.^13^

The KOOS-12 was developed with the intent of being representative of the full KOOS version.^8^ Three subscale scores for pain, function, and QOL can be calculated from the 12-item scale, together with a summary measure of overall knee impact. Item selection and validation was conducted in a large group of patients with knee osteoarthritis. Although KOOS-12 has had limited use in patients with ACL injury, a recent study considered the KOOS-Global and KOOS-12 as equivalent measures in an ACL-reconstructed population due to their high correlation when scores for both scales were calculated at an average of 3 years after surgery.^27^ Although this is a relevant finding, a limitation was that scores were obtained from only 1 timepoint and the responsiveness of each short-form version was not evaluated.

The KOOS-ACL was published in 2023, with items selected and validation occurring in a large cohort of younger patients (age ≤25 years) who underwent ACL reconstruction.^19^ The scale contains 12 items from which 2 subscales (a function subscale with 8 items and a sport subscale with 4 items) are calculated. For both subscales, large mean differences (effect size, >0.8) have been reported between baseline and up to 2 years after ACL reconstruction, with little change thereafter.^18,19^

Given the continued interest in measuring patient-reported outcomes after ACL reconstruction and the benefit of being able to do so at multiple timepoints, short-form scales are highly desirable. However, there is currently a lack of data that compares the responsiveness of these measures at midterm follow-up. Therefore, the purpose of the current study was to determine the responsiveness of 3 short-form versions of the KOOS, the KOOS-12, KOOS-Global, and KOOS-ACL, between 2 and 5 years after ACL reconstruction. It was hypothesized that all versions would have moderate levels of responsiveness.

## Methods

### Participants

The study participants were part of a larger longitudinal study of primary ACL reconstruction that began in December 2013. The larger study was conducted under the auspices of a specialist group of orthopaedic surgeons in a private hospital setting, with 5 surgeons participating in patient recruitment. Inclusion criterion for the longitudinal study was primary ACL reconstruction; exclusion criteria were any surgery to address posterior cruciate ligament injury or treatment of an associated fracture in addition to ACL reconstruction. For the current analysis, patients were eligible if they had completed 2- and 5-year review assessments. Patients who had sustained further ACL injury during the study follow-up period were also excluded, as they were not reviewed routinely at the same timepoints as patients who did not suffer further injury. A total of 683 patients were enrolled in the larger study. Of 334 patients who had follow-up data at the required timepoints, there were 58 patients who were excluded from the analysis due to having sustained a further ACL injury (either graft rupture or contralateral ACL injury), leaving 276 participants included in the current study. The study protocol was approved by the hospital and university ethics committees, and all included participants provided written informed consent.

Regarding surgical technique, arthroscopically assisted surgery was performed using a variety of graft types, but predominately hamstring tendon autograft with suspensory fixation on the femoral side and interference screw fixation on the tibial side. Patients were provided with a standard rehabilitation protocol and guidelines that have been outlined previously.^1^ These encourage immediate full knee extension and the restoration of quadriceps function as soon as possible.^1^ Clearance to return to competitive sport was typically between 9 and 12 months postsurgery and was determined by the treating surgeon^1^.

### Outcome Measures and Procedures

Various demographic (Marx activity scale) and surgical (meniscal and chondral status) characteristics were recorded both preoperatively and during the arthroscopic ACL reconstruction for all patients.^20^ Patients also completed the full 42-item KOOS version at both 2 years (T_1_) and 5 years (T_2_) after ACL reconstruction, and, from this, the following 3 short-form versions were calculated: KOOS-12,^8^ KOOS-Global,^13^ and KOOS-ACL.^19^

The KOOS-Global and KOOS-12 have interval conversions or normalized scoring so that a single score between 0 and 100 is generated, where 100 indicates no symptoms. For KOOS-ACL, the authors of the initial study have noted that calculation of a total score is not appropriate, as it would overrepresent the function scale; however, a composite score (also ranging from 0 to 100) can be calculated by averaging the 2 subscales, and this can be used for clinical interpretability.^19^

At both T_1_ and T_2_, patients were also asked to rate their knee function by responding to the following question: “How would you rate your knee function right now, on a scale from 1 to 100; where 100 is normal?” The data were used to classify patients into 3 groups according to knee function over time: stable (change in scores from T_1_ to T_2_ <5 points), improved (change, >5 point increase), or worsened (change, >5-point decrease).

### Data and Statistical Analysis

Descriptive statistics were calculated. Responsiveness of the 3 KOOS versions was evaluated using both distribution and anchor-based methods.^2,15^ For the distribution method, paired *t* tests were used to compare changes in the various KOOS scores from T_1_ to T_2_, and the standardized response mean (SRM) was calculated by dividing the mean change between T_1_ and T_2_ by the standard deviation (SD) of the mean.^22^ Values >0.8, 0.5, and 0.2 were considered as large, medium, and small, respectively.^4,5^ The standard error of measurement (SEM) was calculated according to the formula $SEM\;=\;SD\times \sqrt{1}-ICC$ , with standard deviation (SD) based on scores from the first assessment and the intraclass correlation coefficient (ICC; 2-way random-type agreement) between both assessments.^23^ The smallest detectable change (SDC) was also calculated, and the following formulas were used to determine the SDC at both individual and group levels: *SDC_individual_* = $1.96\times \sqrt{2}\times SEM$ ; *SDC_group_* = $\left(1.96\times \sqrt{2}\times SEM\right)/\sqrt{n}$ .^3^ As these calculations require a stable sample (ie, no change in knee function), data from the stable knee function group were used.^7^

To determine the minimally important change (MIC) associated with an improvement in knee function, an anchor-based method with a receiver operating characteristic (ROC) curve was used.^28^ The stable and improved knee function groups were used for this analysis. An ROC curve was generated with KOOS change scores as the dependent variable and knee function change status (improved group, stable group) as the independent variable.^4^ After calculating the ROC curve, the diagnostic sensitivity and specificity for each potential MIC value was calculated, and the largest Youden index value (sensitivity + specificity - 1) was used to determine the ideal MIC value.^29^ The area under the ROC curve (AUC) represents the probability that the measure will discriminate correctly between the improved and stable patients; an AUC of 0.5 is random, 0.7 to 0.8 is acceptable, and 0.8 to 0.9 is excellent.^25^ Analyses were repeated for each KOOS version (KOOS-12, KOOS-Global, and KOOS-ACL). Statistical calculations were conducted using IBM SPSS Statistics Version 28 (IBM Corp). For all comparisons, *P* < .05 was considered statistically significant.

## Results

The demographic and surgical characteristics of the 276 study participants are summarized in Table 1. The mean time between the T_1_ and T_2_ was 2.9 ± 0.3 years. All 3 short-form versions were subject to high ceiling effects (Table 2).

**Table 1:** Demographic Characteristics and Surgical Details of Study Participants (N = 276)^*a*^

Characteristic	Value
Age at surgery, y	26.0 ± 8.1
Sex	
Male	149
Female	127
Preinjury Marx activity score	11.9 ± 4.0
Medial meniscal tear	86
No treatment	11
Repair	30
Resect	45^*b*^
Lateral meniscal tear	90
No treatment	44
Repair	7
Resect	39
Chondral damage^*c*^	67
ICRS grade 2	39
ICRS grade 3	20
ICRS grade 4	8
Graft type (all autograft)	
Hamstring tendon	253
Patellar tendon	8
Quadriceps tendon	14
LARS	1
Time after surgery, y	
T_1_	2.3 ± 0.4
T_2_	5.2 ± 0.4
Time between T_2_ and T_2_, y	2.9 ± 0.3
Sport status (N)^*d*^	
No sport	T_1_, 74; T_2_, 63
Training	T_1_, 9; T_2_, 8
Returned to lower level of competition	T_1_, 49; T_2_, 49
Returned to same/higher level of competition	T_1_, 144; T_2_, 156

a Data are reported as mean ± SD or No. of participants. ICRS, International Cartilage Repair Society; T_1_, mean 2-year assessment; T_2_, mean 5-year assessment.

b 4 in a previous surgery.

c Treatment performed in 46% (31/67) of cases of chondral damage (all debrided with an arthroscopic shaver and 1 microfracture procedure). All meniscal and chondral pathology was determined arthroscopically at the time of the reconstruction.

d Sport status was determined by participants checking a box as to whether they had not returned to sport, had returned to training, or had returned to competitive sport (at higher, same, or lower level).

**Table 2:** Percentage of Patients With Maximum Score for KOOS-Global, KOOS-12, and KOOS-ACL^*a*^

	T_1_	T_2_
KOOS-12	17.4	26.8
Pain subscale	45.7	49.6
ADL subscale	64.1	67
QOL subscale^*b*^	19.2	29
KOOS-Global^*b*^	15.6	25.7
KOOS-ACL	25.4	38.4
Function subscale	54.7	59.1
Sport subscale	31.2	45.7

a ACL, anterior cruciate ligament; ADL, activities of daily living; KOOS, Knee injury and Osteoarthritis Outcome Score; QOL, quality of life; T_1_, mean 2-year assessment; T_2_, mean 5-year assessment.

b QOL subscale can also be calculated for KOOS-Global.

KOOS-Global scores increased significantly over time (*P* < .001), whereas KOOS-12 and KOOS-ACL scores did not (Table 3). Of the various subscales, only KOOS-QOL scores increased significantly over time (*P* = .004). Despite statistically significant changes in these measures, the SRM was small for both KOOS-Global and KOOS-QOL (0.26 and 0.17, respectively) (Table 3).

**Table 3:** KOOS-12, KOOS Global, and KOOS-ACL at T_1_ and T_2_ and Difference in Scores^*a*^

	T_1_	T_2_	Δ(T_1_-T_2_)	*P*	SRM
KOOS-12	89.2 ± 10.3	89.8 ± 12.5	0.6 ± 10.1	.3	0.06
Pain subscale	92.6 ± 9.9	92.4 ± 10.6	-0.2 ± 9.6	.7	0.03
ADL subscale	95.8 ± 7.8	95.0 ± 10.4	-0.8 ± 9.2	.2	0.09
QOL subscale^*b*^	79.1 ± 17.5	82.0 ± 19.7	2.9 ± 16.9	**.004**	0.17
KOOS-Global^*b*^	77.0 ± 14.0	80.3 ± 15.6	3.3 ± 12.6	**<.001**	0.26
KOOS-ACL	92.1 ± 9.2	92.4 ± 11.3	0.3 ± 10.4	.7	0.03
Function subscale	96.4 ± 6.2	96.0 ± 8.2	-0.4 ± 7.1	.4	0.05
Sport subscale	87.9 ± 13.7	88.8 ± 15.8	0.9 ± 15.5	.3	0.06

a Data are reported as mean ± SD. ACL, anterior cruciate ligament; ADL, activities of daily living; KOOS, Knee injury and Osteoarthritis Outcome Score; QOL, quality of life; SRM, standardized response mean; T_1_, mean 2-year assessment; T_2_, mean 5-year assessment. Boldface *P* values indicate statistically significant difference between assessments (*P* < .05).

b QOL subscale can also be calculated for KOOS-Global.

Regarding changes in knee function from T_1_ to T_2_, there were 88 patients in the improved group, 115 in the stable group, and 73 in the worsened group. Meniscal status at surgery (intact, torn) or the presence of chondral pathology was not associated with change in knee function (*P* > .05, chi-square test). The SEM and SDC at both individual and group levels for each of the KOOS short-form versions as well as the QOL subscale are shown in Table 4. Values were similar for each of the short-form versions and were slightly higher for the QOL subscale.

**Table 4:** SEM and SDC Values for KOOS-QOL, KOOS-Global, and KOOS-12^*a*^

	SEM	SDC_Individual_	SDC_Group_
KOOS-QOL	5.6	15.6	1.5
KOOS-Global	5.0	13.8	1.3
KOOS-12	3.2	8.8	0.8
KOOS-ACL	3.1	8.6	0.8

a ACL, anterior cruciate ligament; KOOS, Knee injury and Osteoarthritis Outcome Score; QOL, quality of life; SDC, smallest detectable change; SEM, standard error of measurement.

The mean change in KOOS short-form scores for the improved group was 10 to 13 points for KOOS-Global and KOOS-QOL, and, on average, 6 points for KOOS-12 and KOOS-ACL (Table 5). The AUC was acceptable for all measures, and the MIC change value was smallest at 3.2 points for the KOOS-Global and largest at 9.4 points for the KOOS-QOL. For all measures, the difference between the stable and improved groups was greater than the SDC_Group_ and MIC. The KOOS-Global was the only measure for which the mean difference between the 2 assessments for all patients (3.3 points) exceeded both the SDC_Group_ (1.3 points) and MIC (3.2 points).

**Table 5:** MIC Values for the KOOS QOL, KOOS Global, and KOOS 12^*a*^

	Stable Group^*b*^	Improved Group^*c*^	AUC	MIC
KOOS-QOL	2.9 ± 10.4	12.9 ± 14.2	0.72	9.4
KOOS-Global	3.2 ± 9.2	10.2 ± 12.0	0.70	3.2
KOOS-12	0.7 ± 6.4	6.1 ± 7.9	0.73	5.2
KOOS-ACL	0.1 ± 6.4	5.6 ± 8.2	0.71	3.9

a Data are reported as mean ± SD. ACL, anterior cruciate ligament; AUC, area under the curve; KOOS Knee injury and Osteoarthritis Outcome Score; MIC, minimal important change score; QOL, quality of life; T_1_, mean 2-year assessment; T_2_, mean 5-year assessment.

b No change in knee function from T_1_ to T_2_ (n = 115).

c Improved knee function from T_1_ to T_2_ (n = 88).

## Discussion

Of the 3 short-form versions evaluated, KOOS-Global was found to be the only measure that showed a significant score increase over the 3-year follow-up period of the study and the only measure for which the change exceeded the MIC.

The full KOOS scoring system has received some criticism for having limited relevance for ACL reconstructed patients and, with 42 items, has a significant responder burden.^10,26^ The development of short-form versions aims to reduce this burden. The KOOS-12 and KOOS-Global were developed around the same time and published in 2018 and 2019, respectively.^8,13^ The KOOS-ACL has been published only recently.^19^ The KOOS-Global and KOOS-ACL were both developed with, and are intended for use, in ACL-injured populations, whereas the KOOS-12 was developed for a knee osteoarthritis and joint replacement population. Given their relatively recent development, there has been minimal documentation of their use. At the 2- and 5-year postoperative timepoints, all 3 versions were prone to high ceiling effects, particularly the KOOS-ACL. This is of some concern, as it may prevent accurate interpretation of any data collected using such measures.

During the development of both the KOOS-Global and KOOS-ACL, the responsiveness to change was reported as high for both measures between baseline (preoperative) and 2 years.^13,19^ It is perhaps not surprising that large effects are seen when the postoperative state is compared with the preoperative one. It is, therefore, relevant to know how responsive these scales are between postsurgical follow-up timepoints. The current data showed that a small but clinically meaningful increase in score was seen for the KOOS-Global over a mid-term 3-year follow-up period, whereas no change (average difference less than 1 point) occurred for either the KOOS-ACL or KOOS-12. An external validation study for the KOOS-ACL also showed negligible changes between 2 and 6 years for both the function (-0.3 point change) and sport (1.18 points change) subscales using data from the Multicenter Orthopaedic Outcomes Network (MOON) group.^18^

These findings may simply reflect that patients do not change much over this timeframe, and indeed only one-third of the current patient sample reported an overall improvement in their knee function between 2 and 5 years. Nonetheless, if the goal is to detect the subtle changes that do occur during this time, the KOOS-Global was able to do so, whereas the other 2 short-form versions could not. Ceiling effects were lowest for the KOOS-Global and this may also explain why it was the only measure to change over the 2- to 5-year timeframe. That is, there was little scope for improvement in the other 2 measures. Further work is required to understand the responsiveness of these scales in the longer term and whether scores deteriorate if patient function deteriorates or they progress to an osteoarthritic knee.

Although there are minimal data with which to directly compare the current results, the overall KOOS-Global score from the development study was 73.3 at 2 years after ACL reconstruction and is highly consistent with the overall score of 77 currently reported.^13^ KOOS-ACL scores from the external validation study are also highly similar to the current study (composite score of 89.6 at 2 years compared with 92.1 in current study).^18^ This suggests similarity among patient populations and comparable patient responses. Scores for the KOOS-12 were 10 to 12 points higher than the KOOS-Global and were similar to a previous study by our group that showed a high level of correlation between the KOOS-Global and KOOS-12 at an average 3-year follow-up after ACL reconstruction.^27^ We are not aware of any further studies that have utilized the KOOS-12 for an ACL-injured or ACL-reconstructed population to date.

Of the various subscales than can be calculated from the evaluated KOOS short forms, only the QOL subscale could be considered useful for an ACL-reconstructed population at a midterm follow-up. This 4-item subscale is from the full KOOS measure and is included in both the KOOS-Global and KOOS-12. In the current study the KOOS-QOL subscale showed a significant increase between the 2- and 5-year assessments. As the full KOOS is included in many ACL registries, this subscale has been reported frequently in the ACL literature and has often discriminated between different patient groupings. For example, poorer knee-related QOL has been shown to be associated with increased knee laxity and the presence of meniscal injures at surgery.^6,21^ It should be noted that the KOOS-Joint Replacement short form can also be calculated from KOOS-Global, but it was not evaluated in the current study due to previous work showing the presence of high ceiling effects (44% of patients scored 100).^27^ Similarly high ceiling effects were found in the present study for all other subscales of the KOOS-12 and the KOOS-ACL. These subscales should therefore be evaluated with caution.

Short versions of the KOOS have appeal as they can be feasibly implemented and allow for some comparable data with the many ACL registries that utilize the full KOOS measure. However, the availability of multiple short versions does make the choice of which one to select difficult. As previously noted, they have all been developed recently and, as such, there are minimal data on their use and psychometric properties are still being gathered. Therefore, it is currently challenging to make a definite recommendation for one short form over another. Based on our current and previous data,^27^ the KOOS-Global has the best responsiveness and psychometric properties for use at mid-term follow-up after ACL reconstruction compared with other KOOS short forms. However, in research settings, it may be worthwhile to include multiple KOOS short-form versions so that they can be evaluated further. Registry data or data where the full KOOS scale have been used to report psychometric properties in ACL-reconstructed patients could also be utilized to extract KOOS short-form scores so that some long-term data are also available and responsiveness to change further appraised.^11,18^

### Limitations

This study is not without limitations. It is relevant to note that all short-form versions were derived from the full version of the KOOS. It has thus been assumed that patients will respond similarly if presented only with the short-form version. This has, however, been a common methodology that allows accurate head-to-head comparisons between such tools. Patients in the current study were also experiencing routine recovery and had not had significant postoperative complications or reinjuries. They were also recruited via a large metropolitan specialist clinic and were part of an ongoing study. The current patient sample was one of convenience from this larger study. These factors should all be considered when interpreting the generalizability of the patient sample.

## Conclusion

Of the 3 short-form versions of the KOOS that are currently available, the KOOS-Global had the greatest responsiveness to change between 2- and 5-years after ACL reconstruction. High ceiling effects were present for all versions evaluated.
